# Draft Genome Sequence of *Desulfatitalea tepidiphila* S28bF^T^

**DOI:** 10.1128/genomeA.01326-15

**Published:** 2015-11-12

**Authors:** Yuriko Higashioka, Hisaya Kojima, Tomohiro Watanabe, Manabu Fukui

**Affiliations:** The Institute of Low Temperature Science, Hokkaido University, Sapporo, Japan

## Abstract

*Desulfatitalea tepidiphila* S28bF^T^ is a sulfate-reducing bacterium closely related to *Desulfosarcina* species. Here, the draft genome sequence of strain S28bF^T^ is reported.

## GENOME ANNOUNCEMENT

*Desulfatitalea tepidiphila* is a mesophilic sulfate-reducing bacterium belonging to the family *Desulfobacteraceae* within the class *Deltaproteobacteria* (1). The type strain of *D. tepidiphila* is S28bF, isolated from marine sediment obtained from Tokyo Bay in Japan. Strain S28bF^T^ was isolated from a toluene-degrading enrichment culture, but it cannot utilize toluene or other hydrocarbons as an electron donor for growth. The 16S rRNA gene sequence of strain S28bF^T^ shows 93% similarity to the sequence of its closest relative, *Desulfosarcina cetonica* DSM 7267^T^.

Genomic DNA of the strain was extracted using the AquaPure genomic DNA isolation kit (Bio-Rad Laboratories, CA). For next-generation sequencing, an 8-kbp paired-end library was prepared as described previously (2). The library was sequenced using the Genome Sequencer FLX system (Roche Diagnostics), and 437,711 reads were assembled using the GS *De Novo* Assembler version 2.3, with default parameters. The sequences of the gap regions between contigs in the same scaffold were determined by Sanger sequencing of PCR products amplified from genomic DNA or fosmid clones covering the gap regions and then assembled using the Paracel Genome Assembler (Paracel, CA). The resulting assembly comprised 7 contigs totaling 5.6 Mb in size.

*Desulfosarcina* spp. and their relatives are known as sulfate-reducing bacteria capable of degrading various organic substances, and they are regarded as possible key players in carbon dynamics of anaerobic marine environments. The genome data for *Desulfatitalea* spp. will pave the way for better understanding these bacteria with ecologically important functions.

### Nucleotide sequence accession numbers.

This whole-genome shotgun project has been deposited in DDBJ/EMBL/GenBank under the accession no. BCAG00000000. The version described in this paper is the first version, BCAG01000000.
